# Supplementary material for: Exploring the benefits of full-time hospital facility dogs working with nurse handlers in a children’s hospital
Source: PLoS One. 2023 May 31;18(5):e0285768. doi: 10.1371/journal.pone.0285768 (PMC10231821; doi:10.1371/journal.pone.0285768)
Supplement: S1 Fig — This figure is a stacked bar graph created using the likert package in R [27], with the median of the “sometimes” category in the middle and extending to both sides. The numbers on the left side of the bar graph indicate the percentage of healthcare professionals who responded with "“Never” or “Not very often”. The number in the center of the bar graph indicates the percentage of healthcare professionals who responded with “Sometimes”. The numbers on the right side of the bar graph indicate the percentage of healthcare professionals who responded with “Very often” or “Always”. (DOCX) [file pone.0285768.s001.docx]

**S1 Fig. Visualization of the 431 participants’ responses based on the frequency of experiencing the impact of facility dog intervention.** This figure is a stacked bar graph created using the *likert* package in R [27], with the median of the “sometimes” category in the middle and extending to both sides. The numbers on the left side of the bar graph indicate the percentage of healthcare professionals who responded with “Never” or “Not very often”. The number in the center of the bar graph indicates the percentage of healthcare professionals who responded with “Sometimes.” The numbers on the right side of the bar graph indicate the percentage of healthcare professionals who responded with “Very often” or “Always.”
