# Supplementary material for: Exploring the benefits of full-time hospital facility dogs working with nurse handlers in a children’s hospital
Source: PLoS One. 2023 May 31;18(5):e0285768. doi: 10.1371/journal.pone.0285768 (PMC10231821; doi:10.1371/journal.pone.0285768)
Supplement: S1 Table — (DOCX) [file pone.0285768.s002.docx]

**S2 Table. Comparison of the results of nine questions (Q11­-Q19) based on the attributes of the 431 respondents to Q8 “Frequency of accompaniment to interventions”**

χ^2^ df *p* Effect Size[95% CI]

Q11 Patient cooperation 2.19 1 .139 .23 [ -.08 , .50 ]

Q12 Improvement in workload 0.00 1 .961 .01 [ -.31 , .32 ]

Q13 Support for patient decision-making 0.94 1 .333 .27 [ -.33 , .71 ]

Q14 Reduce medication 0.55 1 .459 .16 [ -.29 , .55 ]

Q15 Effects on terminal care 2.53 1 .111 .31 [ -.09 , .62 ]

Q16 Effects on expression 0.00 1 .984 .00 [ -.33 , .33 ]

Q17 Flexibility to schedule change 0.58 1 .448 .20 [ -.35 , .64 ]

Q18 Reduction in verbal abuse and violence 0.10 1 .758 .10 [ -.60 , .72 ]

Q19 Ease of outpatient and readmissions 0.37 1 .543 .14 [ -.35 , .57 ]

**p* < .05. Accepted criteria for effect size *r*: 0.1 (small), 0.3 (medium), 0.5 (large).
