# Supplementary material for: Exploring the benefits of full-time hospital facility dogs working with nurse handlers in a children’s hospital
Source: PLoS One. 2023 May 31;18(5):e0285768. doi: 10.1371/journal.pone.0285768 (PMC10231821; doi:10.1371/journal.pone.0285768)
Supplement: S2 Table — (DOCX) [file pone.0285768.s003.docx]

**S3 Table. All comments made in the free text field of Q12 “Improvement in workload”**

Ward Profession, Comments

Internal medicine children’s ward (age >3 years)

Ns. A patient who had been crying because they did not want to be there for a long time was able

to do their best without crying because of the presence of the facility dog.

Ns. They were able to enter the treatment room smoothly.

Ns. A patient who was reluctant to enter the treatment room was able to go smoothly into the

room with the facility dog.

Ns. A patient was able to engage in procedures and examinations smoothly, and it was a time

saver.

Ns. Patients who needed bone marrow puncture, lumber puncture, or moving into medical operating room without much trouble.

Ns. A patient was able to smoothly move into the treatment room.

Ns. Many patients who initially said “I do not want to do this because it hurts” later said “I can do it with Yogi!” In addition, the care of the children after the treatment was smooth, e.g., they did not cry. They were more confident.

Ns. A patient who was reluctant to undergo examinations and procedures agreed to do so in Yogi’s presence, and the process went smoothly.

Ns. They were able to go to the treatment room promptly.

Ns. When we were pressed for time for an examination, the intervention of the facility dog made it easier for us to go to the examination.

Ns. The patient did not resist as long as before and went smoothly for the examination.

Ns. During bone marrow puncture, lumbar puncture, and other procedures, the facility dog made things easy for us.

Ns. They were able to manage intravenous catheterization more easily during intravenous catheterization.

Pediatric Intensive Care Unit (PICU)

Ns. They were able to tolerate procedures positively without resistance.

Dr. It was easier to perform pain management because they paid attention to Yogi, who was a

good distraction.

Ns. The facility dog often accompanied the patients during anesthesia induction in the operating room. They tried hard because the dog was with them.

Ns. They were able to enter the operating room smoothly.

Ns. Many patients said that they would agree to undergo the examinations and treatment if the

facility dog was with them. Often, they got upset if they were only accompanied by only

nurses and doctors.

Ns. When we went outside the ward for examinations with the facility dog, it was easy as the

patients did not get upset.

Ns. I think it provides mental support but did not make the process any easier, and I think it is hard to expect that kind of impact.

Ns. Yogi’s presence makes them try their best, and the procedures could be started more

smoothly.

Ns. It was a time-saver because the patients did not get upset about the procedures and

examinations.

Ns. Sometimes, patients who had a lot of anxiety or fear about procedures, examinations, or surgery were able to start them more smoothly.

**S3 Table.** (Continued)

Ward Profession, Comments

Surgical Department

Ns. They accompany patients to the operating room and facilitate ambulation.

Ns. No use of sedatives. (Sedatives are not needed if a facility dog is present.)

Dr. They were more cooperative with the intravenous injection and medical treatment as they wanted to be discharged from the hospital as soon as possible and because they thought the facility dog was watching them.

Ns. It made our work more efficient because the patient became more cooperative with the treatment.

Ns. Patients became more cooperative with the procedure.

Ns. It made it easier to get the patient to proceed with the examination and to ensure their

cooperation.

Ns. Patients who were afraid of the procedures got the courage to do their best with the

dog.

Ns. A patient who was reluctant to go to surgery said “I will go if I am with the facility dog!” It was easy to send them to the surgery room.

Dr. Sedation during imaging examinations was no longer necessary.

Cardiac Care Unit (CCU)

Ns. They agreed to do the procedure if the facility dog was with them. It was smoother to start.

Ns. The defiant behavior lasted for a shorter time.

Ns. It became easier to enter the room.

Ns. They got the anesthesia and switched rooms without any reluctance.

Ns. It was easier to do things with the facility dog.

Dr. The ward became brighter.

Outpatient

Ns. It facilitated entry into the surgery room.

Ns. With the facility dog intervention, we were able to do things collaboratively with the patient

who had been refusing to do so earlier.

Ns. The patient who had been afraid of a bone marrow puncture was able to tolerate it with ease.

Ns. The procedure could be performed smoothly without the patient crying or making a fuss.

Ns. I saw them come together to the entrance of the operating room, and the patient seemed

to have gained courage from the facility dog.

Perinatal Centre

Ns. There were occasions when the patients would head to the examinations smoothly when Yogi

or Bailey were there with them.

Ns. A patient who was reluctant to take medication was able to take it when Yogi was having his dog treats on the side. They were able to spend the resting time calmly.

Ns. Patients can go through examinations and procedures smoothly.

Ns. I saw a patient and the facility dog going to radiotherapy together.

Ns. They do not accompany patients for procedures or examinations in West 2 (Perinatal Centre).

Operating rooms

Ns. The patients were able to take the initiative in undergoing the procedure (They were able to go to the treatment room and lie down on the bed without being asked to do so).

Ns. Sedatives were no longer necessary.

Ns. A patient who had refused to go to the operating room said “I will go if I am with Yogi!” and became more positive about it.

**S3 Table.** (Continued)

Ward Profession, Comments

Operating rooms

Dr. They were able to come to the operating room without crying.

Neonatal Intensive Care Unit (NICU) and Growing Care Unit (GCU)

Ns. In terms of Q11 and Q12, it is hard to determine the influence of the facility dog because they are not a practical part of the procedure and examination.

Ns. I felt that the patients received the treatment smoothly when we requested their help both in advance and at the last minute. The word “Yogi” got the patients moving rather than us having to persuade them.

Ns. They were able to smoothly tolerate the examinations they did not like (when I was in North

5: Internal medicine children’s (age >3 years) ward).

Ns. They became more cooperative. The process was faster.

Internal medicine infant ward (age <3 years)

Ns. It took less time to start the procedures with patients who were afraid.

Ns. They did not cry during procedures.

Ns. They tried to do their best during the examinations.

Infection observation ward

Ns. It may not be relevant, but here is my opinion: the facility dog team hardly ever comes to the North 4 ward. The patients want to see them, but they only go to the ICU or the North 5: Internal medicine children’s ward (age >3 years). They seem to go only to the patients with serious illnesses or cancer. Even if we wanted them to come to North 4: Infection observation ward, we could not ask them to because we thought it would be too much for Yogi. At the very least, I would like them to come on some set dates regularly. Even though we let the patients know of the facility dog when they are admitted to the hospital, many of them end up never meeting him.

Ns. They only come to North 4 once in a while, and when they do, they only stop by the playroom or hospital rooms briefly. Hence, we have never had them get involved in examinations or procedures.

Ns. A patient who had been strongly refusing to take the medicine was able to take it immediately after Yogi came. This allowed us to get things done faster and move onto the next shift right away.

Nurse management office

Ns. Patients get less upset.

Ns. It is easier to support the patient’s decision making.

Psychological Treatment for Children and Family

Dr. Patients are less anxious.

Others

Others I felt that the patients spent less time rejecting procedures and examinations, and therefore,

they could be performed smoothly.

Others In some cases, talking about Bailey or Yogi made it easier to engage them in the

conversation.

Others A patient who used to be reluctant to move started to do activities with the facility dog,

including walking practice.

Dr. A patient does not refuse IV drips anymore. They volunteered for the IV.

**S3 Table.** (Continued)

Ward Profession, Comments

Others

Dr. Patients are now able to face surgery

Others Sometimes patients did their best because Yogi was watching them, and at other times, they did not do well because Yogi was not coming.

Others A patient who did not want to come to the examination room can now come with a smile, calling the process as a walk with Yogi.

Others When patients did not like a procedure and resisted or cried, it was often very difficult for the nurses and doctors to calm them down. But the presence of the facility dog takes away that work and allows us to work and perform the procedures more efficiently.

Others It is not a direct impact, but I think having the dog may calm a patient during a procedure.
