# Supplementary material for: Exploring the benefits of full-time hospital facility dogs working with nurse handlers in a children’s hospital
Source: PLoS One. 2023 May 31;18(5):e0285768. doi: 10.1371/journal.pone.0285768 (PMC10231821; doi:10.1371/journal.pone.0285768)
Supplement: S3 Table — (DOCX) [file pone.0285768.s004.docx]

**S4 Table. All comments made in the free text field of Q14 “Reduced medication”**

Ward Profession, Comments

Pediatric Intensive Care Unit (PICU)

Ns. I have seen a number of children who could be induced with anesthesia without pre-medication when the facility dog was with them.

Ns. Use of Ketalar^®^ (Ketamine) before bone marrow puncture. (Ketalar use is not needed if the facility dog is present.)

Dr. Sometimes analgesia can be reduced.

Dr. It helped to distract from the pain during procedures.

Ns. The amount of the PCA bolus decreased during rehabilitation.

Surgical Department

Ns. Patients were able to go into the operating room without premedication. There were less complaints about pain.

Ns. The amount of PCA bolus decreased during the facility dog intervention.

Ns. During bone marrow punctures and lumber punctures, the presence of the facility dog was helpful.

Ns. There were times when I felt that the patient’s anxiety was reduced during the examination.

Operating rooms

Ns. I do not think that the presence of the facility dog reduces the need for premedication for children who need it. I do not think that the amount of medication has changed since the child did not need premedication.

Ns. We did not use the premedication when entering the operating room even though it could have been possible to do so without the facility dog. But I feel that it makes a huge difference on the mental burden of the patients.

Surgical Department/Operating rooms

Dr. Same as response to Q12: Sedation is no longer required during imaging examinations.

Cardiac Care Unit (CCU)

Dr. I have received fewer complaints about pain after the interaction with the dog, but the positive effect of interaction has gone the next day.

Cardiovascular Ward 3/CCU

Dr. There are many younger patients in the cardiovascular ward.

Internal medicine children’s ward (age >3 years)

Ns. During pre-medication before operation, less medication was necessary thanks to the facility dog.

Others

Dr. Premedication upon entry to the operating room is no longer required.

Others During sedation before operation, the presence of the facility dog played a role in reducing medication.

Others The facility dog was helpful during bone marrow punctures, pre and post interventions, reducing fear in patients, stress relief.

PCA, Patient Controlled Analgesia
